# Supplementary material for: Unveiling the transition from niche to dispersal assembly in ecology
Source: Nature. 2023 Jun 7;618(7965):537–42. doi: 10.1038/s41586-023-06161-x (PMC10266978; doi:10.1038/s41586-023-06161-x)
Supplement: Supplementary file 1 — Supplementary Text, Figs. 1–3 and Table 1. [file 41586_2023_6161_MOESM1_ESM.pdf]

nature portfolio

https://doi.org/10.1038/s41586-023-06161-x

Supplementary information

Unveiling the transition from niche to dispersal assembly in ecology

In the format provided by the authors and unedited

Supplementary Information for:

Unveiling the transition from niche to dispersal assembly in ecology

Lynette H. L. Loke[1,*], Ryan A. Chisholm[2]

[1]School of Natural Sciences, Faculty of Science and Engineering, Macquarie University, North Ryde, NSW, 2109, Australia.

[2]Department of Biological Sciences, National University of Singapore, Singapore 177558.

*Corresponding author: lynetteloke@gmail.com

Supplementary Table 1 ———— 2

Supplementary Text and Supplementary Figure 1 ———— 4

Supplementary Figure 2 ———— 6

Supplementary Figure 3 ———— 7

Supplementary Table 1

Supplementary Table 1. List of benthic macroinvertebrate species recorded in this study.

No.	Class	Species
1	Gastropoda	*Drupella margariticola*
2	Gastropoda	*Euplica scripta*
3	Gastropoda	*Pollia fumosa*
4	Gastropoda	*Pardalinops testudinaria*
5	Gastropoda	*Gyrineum natator*
6	Gastropoda	*Tenguella musiva*
7	Gastropoda	*Trochus maculatus*
8	Gastropoda	*Turbo bruneus*
9	Gastropoda	*Monodonta labio*
10	Gastropoda	*Nerita undata*
11	Gastropoda	*Nerita albicilla*
12	Gastropoda	*Nerita chamaeleon*
13	Gastropoda	*Clypeomorus* sp.
14	Gastropoda	*Cerithium* sp.
15	Gastropoda	*Pictocolumbella ocellata*
16	Gastropoda	*Reishia* sp.
17	Gastropoda	*Chicoreus capucinus*
18	Gastropoda	*Semiricinula fusca*
19	Gastropoda	*Cellana radiata*
20	Gastropoda	*Patelloida saccharina*
21	Gastropoda	*Siphonaria atra*
22	Gastropoda	*Siphonaria guamensis*
23	Gastropoda	*Peronia verruculata*
24	Gastropoda	*Astralium calcar*
25	Gastropoda	*Mancinella echinata*
26	Gastropoda	*Jorunna funebris*
27	Polyplacophora	*Acanthopleura gemmata*
28	Bivalvia	*Barbatia amygdalumtostum*
29	Bivalvia	*Isognomon legumen*
30	Bivalvia	*Isognomon isognomum*
31	Bivalvia	*Pectinidae* sp.
32	Bivalvia	*Arca* sp.
33	Bivalvia	*Modiolus* sp.
34	Bivalvia	*Mytilidae* sp.
35	Bivalvia	*Septifer* sp.
36	Malacostraca	*Metopograpsus* sp.
37	Malacostraca	*Nanosesarma minutum*
38	Malacostraca	*Dardanus megistos*
39	Malacostraca	*Ocypodoidea* sp.
40	Malacostraca	*Dromiidae* sp.
41	Malacostraca	*Diogenes* sp.

42	Malacostraca	*Clibanarius* sp.
43	Malacostraca	*Ligia exotica*
44	Malacostraca	*Amphipoda* sp.
45	Malacostraca	*Alpheus* sp.
46	Malacostraca	*Porcellanidae*
47	Thecostraca	*Balanus* sp.
48	Thecostraca	*Tetraclita* sp.
49	Thecostraca	*Chthamalus* sp.
50	Polychaeta	*Serpulidae* sp.
51	Polychaeta	*Vermetidae* sp.
52	Polychaeta	*Sabellidae* sp.
53	Polychaeta	*Nereididae* sp.
54	Polychaeta	*Eunice* sp.
55	Polychaeta	*Polycladida* sp.
56	Polychaeta	*Branchiomma* sp.
57	Ophiuroidea	*Ophiuroidea* sp.
58	Ascidiacea	*Polycarpa* sp.
59	Ascidiacea	*Didemnum psammatodes*
60	Ascidiacea	*Ascidia gemmata*
61	Demospongiae	*Mycale* sp.
62	Demospongiae	*Porifera* sp.
63	Demospongiae	*Amorphinopsis excavans*
64	Hexacorallia	*Zoanthus* sp.

Supplementary Text and Supplementary Figure 1

Abiotic conditions within setups and surrounding seawall

Prior to the experiment, we conducted trials to test our experimental setups during which we checked for consistency of temperature and light levels within the setups. Temperature loggers (HOBO MX220) were used to record light and temperature data inside and outside the low ($h = 5$) and median ($h = 15$) treatment setups over one month. The logger inside the setup was attached to the surface of the concrete tile while the logger outside the setup was attached directly onto the seawall beside the setup. There were minimal differences in temperature and light levels (average absolute temperature and light level difference was 0.21 °C and 42.97 lux in the $h = 5$ treatment and 0.18 °C and 15.54 lux in the $h = 15$ treatment; Supplementary Fig. 1). We did not measure the potential influence of other variable such as pH, calcium content or material type as it has been established in a previous studies[1–3,] , conducted at several locations worldwide, including one that was approximately 1 km from our present study location along the same shoreline, that differences in pH and material type have no measurable effect on the species richness of the experimental seawall communities we studied.

We also conjecture that if there were substantial variation in unmeasured abiotic conditions, the tendency would be for these conditions to be more stressful in the low-immigration treatments which would tend to lower species richness in these treatments (relative to the trend extrapolated from the high-immigration treatments). Any resulting systematic error would thus tend to work against the novel hypothesis, by making the richness versus immigration curve look more like Fig. 1c than Fig. 1d.

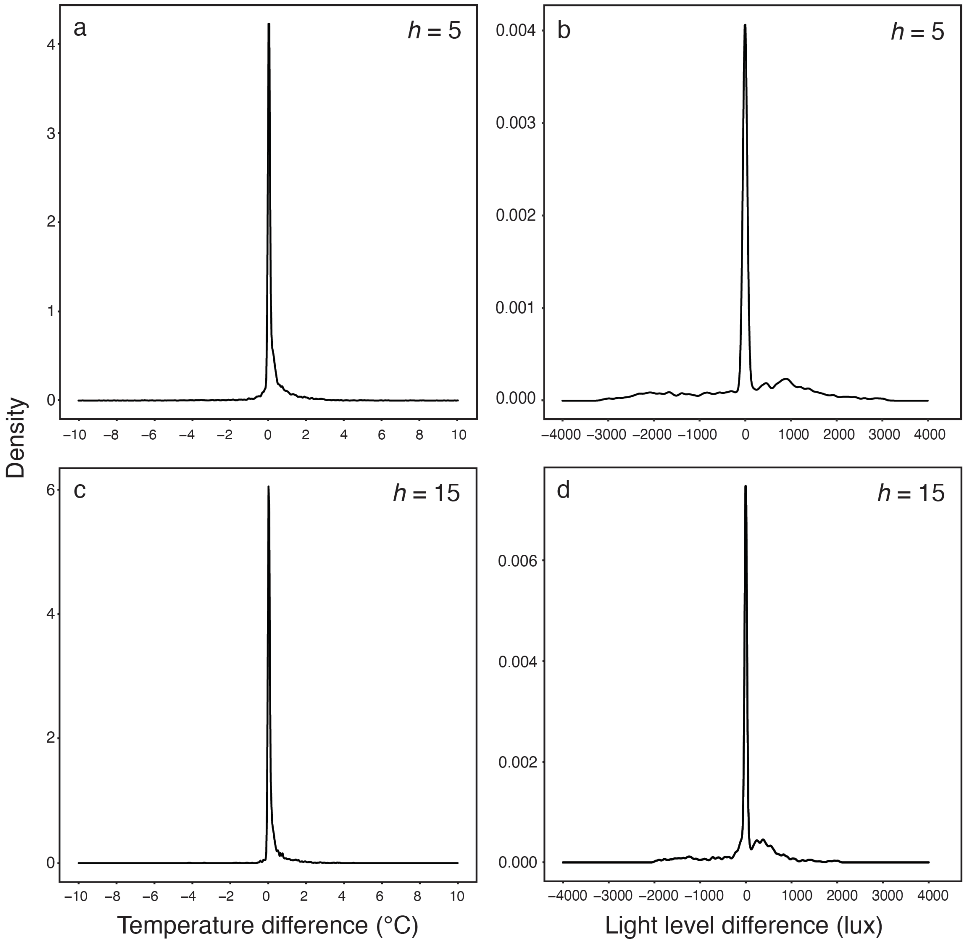

Supplementary Fig. 1. Kernel density estimation of the differences in temperature (°C) and light levels (lux) inside and outside the low ($h = 5$, panels a, b) and median ($h = 15$, panels c, d) experimental setups.

References

1. Hartanto, R. S. et al. Material type weakly affects algal colonisation but not macrofaunal community in an artificial intertidal habitat. *Ecol. Eng.* **176**, 106514 (2022).
2. Dodds, K. C. et al. Material type influences the abundance but not richness of colonising organisms on marine structures. *J. Environ. Manage.* **307**, 114549 (2022).
3. Hsiung, A. R. et al. Little evidence that lowering the pH of concrete supports greater biodiversity on tropical and temperate seawalls. *Mar. Ecol. Prog. Ser.* **656**, 193–205 (2020).

Supplementary Figure 2

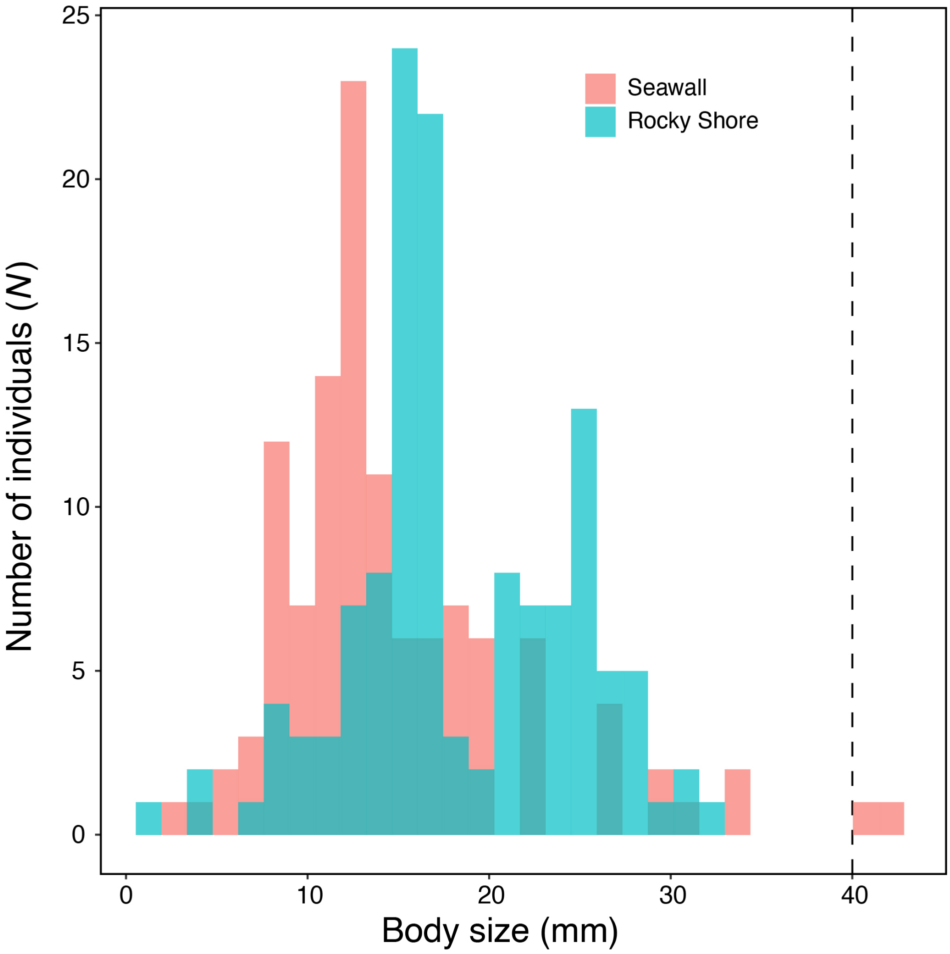

Supplementary Fig. 2. Distribution of maximum body size across species in intertidal communities near our study site. Data are taken from seawalls and rocky shores within approximately 1 km of our study site. Two individuals of the 245 recorded had maximum body lengths > 40 mm (indicated by the vertical dashed line); their widths were less than 40 mm.

Supplementary Figure 3

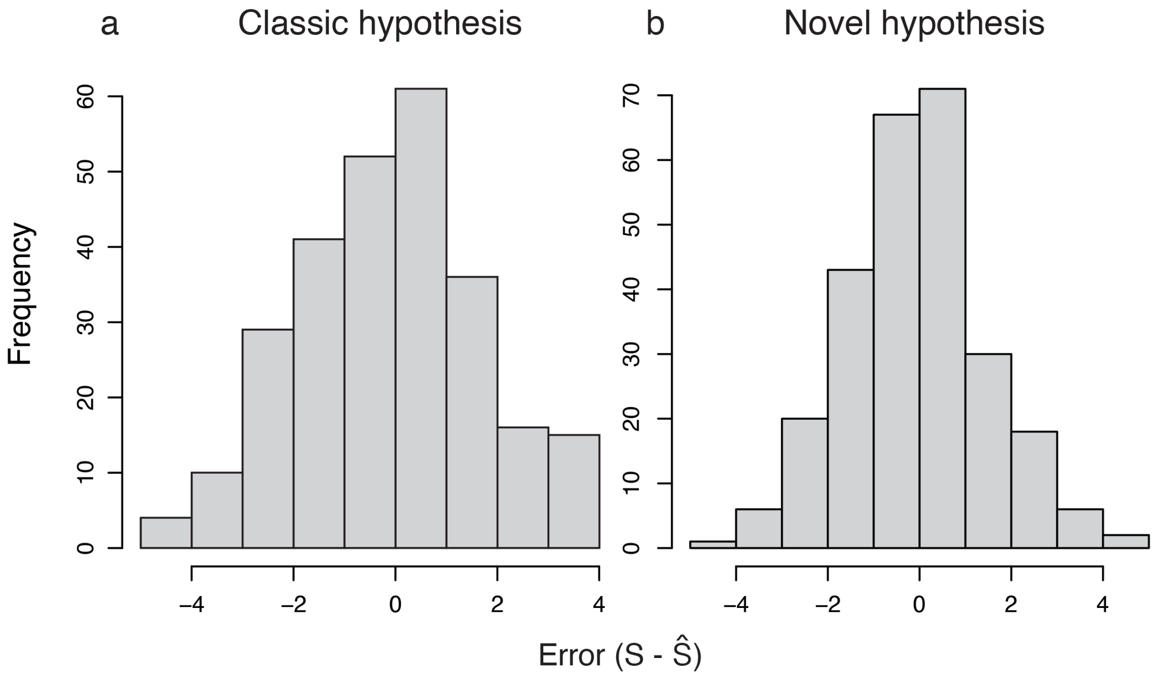

Supplementary Fig. 3. Distribution of the model errors. Errors (observed S – fitted S) for (a) the classic model, and (b) the novel model.
