# Supplementary material for: Unveiling the transition from niche to dispersal assembly in ecology
Source: Nature. 2023 Jun 7;618(7965):537–42. doi: 10.1038/s41586-023-06161-x (PMC10266978; doi:10.1038/s41586-023-06161-x)
Supplement: Supplementary file 2 — Reporting Summary [file 41586_2023_6161_MOESM2_ESM.pdf]

nature portfolio

Corresponding author(s): Lynette H. L. Loke

Last updated by author(s): Apr 13, 2023

Reporting Summary

Nature Portfolio wishes to improve the reproducibility of the work that we publish. This form provides structure for consistency and transparency in reporting. For further information on Nature Portfolio policies, see our Editorial Policies and the Editorial Policy Checklist.

Statistics

For all statistical analyses, confirm that the following items are present in the figure legend, table legend, main text, or Methods section.

n/a	Confirmed	
☐	☒	The exact sample size (*n*) for each experimental group/condition, given as a discrete number and unit of measurement
☐	☒	A statement on whether measurements were taken from distinct samples or whether the same sample was measured repeatedly
☐	☒	The statistical test(s) used AND whether they are one- or two-sided<br>*Only common tests should be described solely by name; describe more complex techniques in the Methods section.*
☒	☐	A description of all covariates tested
☐	☒	A description of any assumptions or corrections, such as tests of normality and adjustment for multiple comparisons
☐	☒	A full description of the statistical parameters including central tendency (e.g. means) or other basic estimates (e.g. regression coefficient) AND variation (e.g. standard deviation) or associated estimates of uncertainty (e.g. confidence intervals)
☒	☐	For null hypothesis testing, the test statistic (e.g. *F*, *t*, *r*) with confidence intervals, effect sizes, degrees of freedom and *P* value noted<br>*Give P values as exact values whenever suitable.*
☒	☐	For Bayesian analysis, information on the choice of priors and Markov chain Monte Carlo settings
☒	☐	For hierarchical and complex designs, identification of the appropriate level for tests and full reporting of outcomes
☐	☒	Estimates of effect sizes (e.g. Cohen's *d*, Pearson's *r*), indicating how they were calculated

Our web collection on statistics for biologists contains articles on many of the points above.

Software and code

Policy information about availability of computer code

Data collection	No software was used for data collection in this study.
Data analysis	All data analyses and figures were generated using R software version 3.5.1 as described in the Methods. Rhino software version 6 was used to design experimental tile in this study. All code used to analyse the data in this study are available in the the Zenodo repository at: 10.5281/zenodo.7819940 (see also 'Code availability' section).

For manuscripts utilizing custom algorithms or software that are central to the research but not yet described in published literature, software must be made available to editors and reviewers. We strongly encourage code deposition in a community repository (e.g. GitHub). See the Nature Portfolio guidelines for submitting code & software for further information.

Data

Policy information about availability of data

All manuscripts must include a data availability statement. This statement should provide the following information, where applicable:

- Accession codes, unique identifiers, or web links for publicly available datasets
- A description of any restrictions on data availability
- For clinical datasets or third party data, please ensure that the statement adheres to our policy

The data underlying this study are available in the Zenodo repository at: 10.5281/zenodo.7819940

Research involving human participants, their data, or biological material

Policy information about studies with human participants or human data. See also policy information about sex, gender (identity/presentation), and sexual orientation and race, ethnicity and racism.

Reporting on sex and gender	n/a
Reporting on race, ethnicity, or other socially relevant groupings	n/a
Population characteristics	n/a
Recruitment	n/a
Ethics oversight	n/a

Note that full information on the approval of the study protocol must also be provided in the manuscript.

Field-specific reporting

Please select the one below that is the best fit for your research. If you are not sure, read the appropriate sections before making your selection.

☐ Life sciences ☐ Behavioural & social sciences ☒ Ecological, evolutionary & environmental sciences

For a reference copy of the document with all sections, see nature.com/documents/nr-reporting-summary-flat.pdf

Ecological, evolutionary & environmental sciences study design

All studies must disclose on these points even when the disclosure is negative.

Study description	We conducted a manipulative field experiment involving the installation of 60 experimental setups or units (12 treatments × 5 replicates) on intertidal seawalls in Singapore (for more details please refer to the main text and Methods).
Research sample	The number of species, species identities, and abundances of the macrofaunal intertidal communities within each setup were censused every month for a year. Major taxa included gastropods, bivalves, polychaetes, tunicates and crustaceans (please refer to the Supplementary information for a full list of the benthic macroinvertebrate species recorded in this study).
Sampling strategy	Sample size selection was based on findings from previous studies done at the same study site and nearby locations, and determined by logistical feasibility (for more details please refer to the main text and Methods).
Data collection	Number of species, species identities, and abundances of the macrofaunal intertidal communities within each setup were censused by the lead and corresponding author every month for a year.
Timing and spatial scale	The study was conducted from from March 2021 to March 2022. Experimental setups were installed in randomised order along the base of intertidal seawalls across a 400 m stretch at our study site in Singapore, and maintained every 2–4 weeks during low tide.
Data exclusions	None.
Reproducibility	Specifications of our experimental setups and fabrication details are provided. Data and code to reproduce the analyses of the empirical data are available.
Randomization	All experimental setups were installed in randomised order along the base of intertidal seawalls at our study site.
Blinding	All setups were censused.

Did the study involve field work? ☒ Yes ☐ No

Field work, collection and transport

Field conditions	Field work was conducted during low tide in clear weather conditions. For the duration of the study, the average reported temperature was 27.9°C and the average monthly precipitation was 19.6 cm at our study site.
Location	The study was conducted along the base of intertidal seawalls (~0.5 m above chart datum) specifically at 1°15'00.4"N, 103°49'04.0" E in Singapore.

Access & import/export	Research permits from the Singapore National Parks Board (NP/RP20-077) were obtained to conduct the research. No specimens were collected or removed from the field. We notified all relevant officer(s) at Sentosa Development Corporation before entering our study site to conduct the research.
Disturbance	Disturbance was minimal and all experimental setups were removed at the end of the experiment.

Reporting for specific materials, systems and methods

We require information from authors about some types of materials, experimental systems and methods used in many studies. Here, indicate whether each material, system or method listed is relevant to your study. If you are not sure if a list item applies to your research, read the appropriate section before selecting a response.

Materials & experimental systems

n/a	Involved in the study	
☒	☐	Antibodies
☒	☐	Eukaryotic cell lines
☒	☐	Palaeontology and archaeology
☐	☒	Animals and other organisms
☒	☐	Clinical data
☒	☐	Dual use research of concern
☒	☐	Plants

Methods

n/a	Involved in the study	
☒	☐	ChIP-seq
☒	☐	Flow cytometry
☒	☐	MRI-based neuroimaging

Animals and other research organisms

Policy information about studies involving animals; ARRIVE guidelines recommended for reporting animal research, and Sex and Gender in Research

Laboratory animals	The study did not involve laboratory animals.
Wild animals	Benthic macroinvertebrates (mainly gastropods, bivalves, polychaetes, tunicates and crustaceans; age unknown) were counted in the field and not captured or removed.
Reporting on sex	n/a
Field-collected samples	No specimens were collected or removed from the field.
Ethics oversight	None required.

Note that full information on the approval of the study protocol must also be provided in the manuscript.
